# Cardiovascular risk factors among patients with human immunodeficiency viral infection at a tertiary hospital in Ghana: a cross-sectional study

**DOI:** 10.11604/pamj.2021.38.317.28335

**Published:** 2021-03-30

**Authors:** Olutobi Adekunle Sanuade, Leonard Baatiema, Aaron Kobina Christian, Peter Puplampu

**Affiliations:** 1 Institute for Global Health, University College London, 30 Guilford Street, London, WC1N 1EH, United Kingdom,; 2 Department of Health Policy, Planning and Management, School of Public Health, University of Ghana, Legon, Accra, Ghana,; 3 Regional Institute for Population Studies (RIPS), University of Ghana, Legon, Accra, Ghana,; 4 Department of Medicine, School of Medicine and Dentistry, College of Health Sciences, University of Ghana, Accra, Ghana

**Keywords:** HIV, cardiovascular risk factors, People Living with HIV/AIDs, Korle Bu Teaching Hospital, Ghana

## Abstract

**Introduction:**

the provision of antiretroviral treatment (ART) to people living with HIV/AIDS (PLHIV) has improved their life expectancy significantly. Conversely, this has been associated with an elevated risk of cardiovascular diseases. Yet, research to improve understanding of cardiovascular risk factors among PLHIV remains limited. This study examines the prevalence and correlates of cardiovascular risk factors among PLHIV at the Korle Bu Teaching Hospital (KBTH) in Accra, Ghana.

**Methods:**

a cross-sectional study was conducted at the KBTH, Accra, Ghana. Patients were recruited from the adult HIV outpatient clinic at the infectious disease unit, KBTH. The sample comprised 525 PLHIV, aged 18 years and above. Data were analysed using descriptive statistics and a multivariable binary logistic regression.

**Results:**

among the patients, 9.7% (n=51) had hypertension and 15.6% (n=82) were reportedly patients with diabetes. With respect to the serum lipid profile, 24.8% (n=130) had hypertriglyceridemia, 49.1% (n=258) had hypercholesterolemia, 26.3% (138) had low high-density lipoprotein, and high low-density lipoprotein was found in 27.2% (n=143) of the cohort. The multivariable binary logistic regression results showed that being unemployed, underweight, being on ART, being male, having a higher level of education, and not having health insurance subscription significantly increased the odds of cardiovascular risk factors among the patients.

**Conclusion:**

current findings buttress concern for elevated risk of cardiovascular diseases among PLHIV and calls for increased attention for comprehensive care that includes the prevention and management of cardiovascular diseases and its risk factors among this vulnerable group.

## Introduction

Despite progress made in human immunodeficiency viral infection (HIV) prevention and treatment, it continues to be a major public health priority globally. At the end of 2019, about 38 million people were living with HIV and over two-thirds (25.7 million) live in the African region [1]. Increased access to antiretroviral therapy (ART) has become a topmost priority as evidence showed that the management of HIV with ART has already significantly reduced HIV/AIDS attributable mortality and incidence of some opportunistic diseases [2]. Concurrently, non-communicable diseases (NCDs) including cardiovascular diseases, cancer, diabetes, among others are now major causes of morbidity and mortality and are projected to overtake the burden of infectious diseases by 2030 [3].

To date, significant proportion of people living with HIV/AIDs (PLHIV) are living with NCDs [4]. For instance, a systematic review and meta-analysis of 194 studies showed that the global prevalence of hypertension among PLHIV is 23.6%. It was estimated that in 2018, there were 8.9 million cases of hypertension among the global population of PLHIV with 59.2% of these individuals living in sub-Saharan Africa [5]. Also, research showed a high prevalence of elevated total cholesterol (24.2%), low HDL cholesterol (53.7%), elevated LDL cholesterol (20.8%), and elevated triglyceride concentrations (22.7%) among African adults living with HIV [6]. This situation is attributed to different reasons including the HIV infection, antiretroviral therapy, increasing age of PLHIV, HIV related inflammation and the demographic and epidemiologic transitions [7]. A large body of research shows that PLHIV and on ART has an increased risk of cardiovascular events [6, 8], depression [9], cancers [10], metabolic abnormalities, and diabetes [11-13]. Consequently, among treated HIV-infected patients, NCD conditions (not linked with the HIV infection) have increasingly been contributing to their morbidity and mortality [14]. Evidence further demonstrates that HIV infection is by itself associated with dyslipidaemia, atherosclerosis and type 2 diabetes (T2D) via chronic inflammation and immune dysfunction mechanisms [15]. The use of ART is also linked with the redistribution of body fat, hypertension, dyslipidaemia, insulin resistance, and dysglycaemia [16].

In Ghana, although well-established evidence exists on the burden of cardiovascular risk factors or HIV, evidence on the burden of cardiovascular risk factors among people living with HIV is highly under-researched, limited and poorly understood despite increasingly being recognized as a major public health threat. In Ghana, only a few studies have advanced our understanding of the cardiovascular risk-HIV comorbid epidemiological burden [17, 18]. Although these studies have provided important insights on the risks of comorbid conditions among PLHIV, they are limited in scope and geographical coverage. For instance, these studies mainly focus on prevalence and determinants of hypertension, hypercholesterolemia and diabetes and have been conducted at one health facility. Studies from other health facilities in other parts of the country will offer an increased understanding of the problem to ensure the development of well-tailored interventions to improve healthcare delivery and overall patient outcomes. Using data from the adult HIV outpatient clinic at the Korle Bu Teaching Hospital (KBTH) in Accra, Ghana, we examined the prevalence and factors associated with cardiovascular risk factors (hypertension, diabetes, hypercholesterolemia, low high-density liproprotein, elevated low-density lipoprotein, and hypertriglyceridemia) among PLHIV.

## Methods

**Study design, sample and sampling strategy:** this cross-sectional study was conducted at the Korle Bu Teaching Hospital, Accra, Ghana. Patients were recruited from the adult HIV outpatient clinic at the infectious disease unit, KBTH. Korle Bu Teaching Hospital is the largest tertiary hospital in Ghana and has more than 2000 bed capacity and an outpatient department attendance of over 350,000 in 2016 [19]. At the time this study was conducted, about 19,000 people living with HIV (PLHIV) was attending the infectious disease unit. Out of this number, 7000 were on antiretroviral therapy (ART). The sample comprised 525 PLHIV aged 18 years and above. Participants were recruited through a systematic random sampling. The first patients who attended the clinic each day were recruited into the study until the total sample was achieved. None of the patients enrolled for this study declined consent. Details of the sample size determination has been provided elsewhere [19].

**Data collection:** respondents were recruited from patients attending the outpatient clinic of the infectious disease unit. Data was collected by a trained research assistant through questionnaire. Data collected included: socio-demographic characteristics: gender, age, level of education, employment status, monthly household income, and ethnicity; lifestyle: alcohol intake and smoking status; anthropometric measurements: height, weight, hip girth, and waist girth.

**Laboratory evaluation:** fasting blood samples were collected from each patient after a 12-h overnight fast. Analysis was carried out at a local commercial laboratory facility. Serum samples were evaluated for lipoprotein (total cholesterols, LDL-low-density lipoprotein, HDL-high-density lipoprotein, and triglycerides), lactate, haemoglobin, and fasting glucose. CD4 leukocytes and neutrophilis were also counted.

**Measures:** HIV diagnosis was made at the infectious unit by antibody detection on blood samples followed by laboratory confirmation using the OraQuick test according to the Ghana National AIDS Control Programme guidelines [19]. A participant was declared positive for HIV if both tests were positive. Three blood pressure measurements were taken from consenting patients; hypertension was therefore defined as an average systolic blood pressure (SBP) ≥ 140mm Hg and/or an average diastolic blood pressure (DBP) ≥ 90mm Hg of the three readings. Diabetes was defined as having a fasting blood sugar ≥ 126mg/dl. Hypercholesterolemia was defined as total cholesterol ≥ 200mg/dL [17]. Low High-density lipoprotein (HDL) was measured as < 40mg/dL for men or < 50mg/dl for women [17]. Elevated LDL was defined as ≥160mg/dL. Finally, we defined hypertriglyceridemia as triglycerides ≥ 150mg/dL [17]. Body Mass Index (BMI) of less than 18.5kg/m^2^ was defined underweight, 18.5 to 24.9kg/m^2^ was defined normal body weight, 25 to 29.9kg/m^2^ was defined overweight, and ≥ 30kg/m^2^ was defined obesity [17].

**Data analysis:** descriptive statistics such as frequency distributions were used to describe the socio-demographic, BMI status and lifestyle characteristics of the study respondents. Univariable analysis was performed using binary logistic regression. All variables (whether significant at the univariable level or not) were included in the multivariable analysis because of their theoretical importance to cardiovascular risk factors. Multivariable binary logistic regressions were used to examine the correlates of hypertension, diabetes and serum lipids and the alpha level of statistical significance was set at 0.05. Data were analyzed using STATA 14.

**Ethical considerations:** ethical clearance and permission was sought and approved by the Ethical and Protocol Review Committee of the University of Ghana Medical School, and the Protocol Identification Number was MS-Et/M.9-P 5.7/2012-13 [19]. Respondents were informed about the objectives of the study, and written informed consent was obtained from all participating individuals before the commencement of the study. Data for this study were stored according to KBTH´s policy and were de-identified to protect patients´ privacy. The data obtained from this study are available and can be shared for justified purposes upon specific request and in accordance with the data management regulations of the College of Health Sciences in Ghana [19].

## Results

**Socio-demographic profiles:** five hundred and twenty-five patients (525) living with HIV were recruited for this study and majority were males (84.4%; n=437) (Table 1). Their mean age was 33.6 (SD=5.0) years. Their age ranged from 19 to 40 years and more than eighty percent (n=419) were between 30-40 years. Almost half (49.9%; n=262) had elementary education and the least proportion (9.5%; n=50) had tertiary education. About eight out of ten of the patients (79.5%; n=403) were employed. While the monthly household income of close to half (46.3%; n=224) of the patients was less than GHS 200.00, slightly more than one-fifth (22.9%; n=111) earned more than GHS 1000.00 per month, and the least proportion (2.5%; n=12) earned between GHC 800-1000 per month. About forty-one percent (n=212) of the patients were Akan, Ewe (18.5%; n=95), Ga Adangbe (12.1%; n=62) and 28.2% (n=145) belonged to other ethnic groups. The average time for individuals having been diagnosed with HIV was 3.1 years; close to 80% (n=396) had been living with HIV for 0-5 years, 18.5% (n=93) for 6-10 years, and 2.8% (n=14) for 11 years or more. About 2% (n=10) were current smokers, 10.0% (n=52) were current consumers of alcohol, and slightly more than one-third (33.8%; n=167) were overweight/obese. About 60.0% (314) were currently undergoing ART.

**Table 1:** socio-demographic, BMI and lifestyle characteristics of study respondents

Characteristic	Total (n=525)	%
**Gender**		
Male	437	84.4
Female	81	15.6
**Age in years**		
19 - 29	99	19.1
30 - 40	419	80.9
**Level of education**		
None	88	16.8
Elementary	262	49.9
Secondary	125	23.8
Tertiary	50	9.5
**Employment status**		
Unemployed	104	20.5
Employed	403	79.5
**Monthly household income (GHS)**		
< 200	224	46.3
200 - 399	81	16.7
400 - 599	39	8.0
600 - 799	17	3.5
800 - 1000	12	2.5
> 1000	111	22.9
**Ethnicity**		
Akan	212	41.2
Ewe	95	18.5
Ga Adangme	62	12.1
Others	145	28.2
**Insurance status**		
No insurance	128	25.0
Have insurance but do not use	237	46.3
Have insurance and use it	147	28.7
**Length of HIV status in years**		
0-5	396	78.7
6-10	93	18.5
11+	14	2.8
**Smoking status**		
Non-smokers	512	98.1
Smokers	10	1.9
**Alcohol consumption**		
Current consumers	52	10.0
Non-consumers	469	90.0
**BMI status**		
Underweight	60	12.2
Normal	266	54.0
Overweight	115	23.3
Obese	52	10.5
**Current ART status**		
No	211	40.2
Yes	314	59.8

**Prevalence of cardiovascular risk factors:** among the patients, 9.7% (n=51) had hypertension and 15.6% (n=82) had diabetes (Table 2). Also, 24.8% (n=130) had hypertriglyceridemia, 49.1% (n=258) had hypercholesterolemia, 26.3% (n=138) had low high-density lipoprotein, and 27.2% (n=143) had elevated low-density lipoprotein.

**Table 2:** prevalence of cardiovascular risk factors

NCDs	Total (n=525)	%
**Hypertension**		
Yes	51	9.7
No	474	90.3
**Diabetes**		
Yes	82	15.6
No	443	84.4
**Triglycerides**		
High	130	24.8
Low	395	75.2
**Cholesterol**		
High	258	49.1
Low	267	50.9
**HDL**		
Low	138	26.3
High	143	73.7
**LDL**		
High	143	27.2
Low	382	72.8

**Correlates of cardiovascular risk factors:** multivariable logistic analysis showed that the correlates of diabetes among the HIV patients included employment status, BMI, and current ART status (Table 3). Patients who were employed had lower odds of having diabetes compared to those who were unemployed [adjusted Odds Ratio (aOR) (95% CI): 0.257 (0.070 - 0.947); P= 0.041]. Those with normal BMI were 64.1% less likely to have diabetes compared to who were underweight [aOR (95% CI): 0.359 (0.160 - 0.806); P= 0.013]. The odds of having diabetes was higher among those who were currently undergoing ART [aOR (95% CI): 2.178 (1.114 - 4.257); P= 0.023]. The results further showed that the odds of having hypertriglyceridemia were higher for those with tertiary education [aOR (95% CI): 3.093 (1.170 - 8.179); P= 0.023] and those currently undergoing ART [aOR (95% CI): 2.389 (1.331 - 4.287); P=0.004], compared to their respective counterparts. However, those who were employed had lower odds of having hypertriglyceridemia compared to those who were employed [aOR (95% CI): 0.340 (0.122 - 0.944); P= 0.038].

**Table 3:** univariable and multivariable predictors of cardiovascular risk factors

	Univariable Analysis		Multivariable Analysis	
	OR (95% CI)	p Value	OR (95% CI)	p Value
**Diabetes**				
**Employment status (**ref=unemployed)				
Employed	0.813 (0.462 - 1.433)	0.475	0.257 (0.070 - 0.947)	0.041
**BMI status** (ref=underweight)				
Normal	0.455 (0.229 - 0.901)	0.024	0.359 (0.160 - 0.806)	0.013
Overweight	0.594 (0.277 - 1.274)	0.181	0.474 (0.192 - 1.169)	0.105
Obese	0.467 (0.174 - 1.253)	0.130	0.426 (0.137 - 1.330)	0.142
**ART medication status (**ref=not on ART)				
Currently on ART medication	1.891 (1.127 - 3.173)	0.016	2.178 (1.114 - 4.257)	0.023
**Hypertriglyceridemia**				
**Level of education (**ref=no education)				
Elementary	0.739 (0.431 - 1.268)	0.272	0.751 (0.396 - 1.426)	0.381
Secondary	0.509 (0.266 - 0.975)	0.042	0.495 (0.226 - 1.085)	0.079
Tertiary	1.590 (0.768 - 3.292)	0.212	3.093 (1.170 - 8.179)	0.023
**Employment status (**ref=unemployed)				
Employed	0.713 (0.444 - 1.147)	0.163	0.340 (0.122 - 0.944)	0.038
**ART medication status (**ref=not on ART)				
Currently on ART medication	1.979 (1.288 - 3.041)	0.002	2.389 (1.331 - 4.287)	0.004
**Hypercholesterolemia**				
**Gender (**ref= male)				
Female	0.458 (0.278 - 0.755)	0.002	0.492 (0.260 - 0.933)	0.030
**Ethnicity (**ref= Akan)				
Ewe	1.685 (1.034 - 2.746)	0.036	2.333 (1.276 - 4.265)	0.006
Ga Adangme	1.456 (0.825 - 2.570)	0.195	1.337 (0.679 - 2.633)	0.400
Others	1.334 (0.873 - 2.037)	0.183	1.588 (0.916 - 2.751)	0.099
**ART medication status (**ref=not on ART)				
Currently on ART medication	3.992 (2.746 - 5.802)	0.000	4.467 (2.702 - 7.385)	0.000
**High LDL**				
**Gender (**ref= male)				
Female	0.415 (0.218 - 0.793)	0.008	0.374 (0.164 - 0.854)	0.019
**Level of education (**ref=no education)				
Elementary	1.030 (0.603 - 1.757)	0.915	1.071 (0.572 - 2.006)	0.830
Secondary	0.568 (0.297 - 1.086)	0.087	0.486 (0.223 - 1.060)	0.070
Tertiary	1.545 (0.740 - 3.222)	0.247	3.837 (1.430 - 10.296)	0.008
**Ethnicity (**ref= Akan)				
Ewe	1.357 (0.783 - 2.351)	0.277	1.581 (0.828 - 3.021)	0.165
Ga Adangme	1.750 (0.945 - 3.242)	0.075	2.010 (1.011 - 4.363)	0.047
Others	1.488 (0.923 - 2.401)	0.103	1.956 (1.083 - 3.532)	0.026
**ART medication status (**ref=not on ART)				
Currently on ART medication	2.636 (1.713 - 4.057)	0.000	2.936 (1.661 - 5.189)	0.000
**Low HDL**				
**Gender (**ref= male)				
Female	5.987 (3.622 - 9.900)	0.000	5.632 (2.724 - 10.874)	0.000
**Employment status (**ref=unemployed)				
Employed	1.098 (0.669 - 1.803)	0.711	3.329 (1.065 - 10.404)	0.039
**Health insurance status (**ref= no insurance)				
Have insurance but do not use	0.383 (0.240 - 0.608)	0.000	0.582 (0.276 - 0.990)	0.047
Have insurance and use it	0.285 (0.165 - 0.494)	0.000	0.373 (0.179 - 0.776)	0.008
**BMI status** (ref=underweight)				
Normal	0.495 (0.277 - 0.881)	0.017	0.582 (0.265 - 1.275)	0.176
Overweight	0.327 (0.164 - 0.649)	0.001	0.363 (0.145 - 0.907)	0.030
Obese	0.274 (0.113 - 0.661)	0.004	0.344 (0.104 - 1.135)	0.080
**ART medication status (**ref=not on ART)				
Currently on ART medication	0.145 (0.094 - 0.225)	0.000	0.121 (0.061 - 0.238)	0.000

BMI= body mass index; CI=confidence interval; OR=odds ratio; ART=antiretroviral therapy; LDL= low-density lipoprotein; HDL=high-density lipoprotein. Only variables significant at the multivariable analysis were reported. Variables included in the models include gender, age, level of education, employment status, income, ethnicity, health insurance status, length of HIV status, smoking status, alcohol consumption status, BMI status, and ART status. None of the variables were significant predictors of hypertension

Regarding cholesterol level, while females had lower odds of having hypercholesterolemia, those who were Ewe [aOR (95% CI): 2.333 (1.276 - 4.265); P= 0.006] and those currently undergoing ART [aOR (95% CI): 4.467 (2.702 - 7.385); P= 0.000] had higher odds of having hypercholesterolemia compared to their respective counterparts. The predictors of elevated LDL include gender, level of education, ethnicity and ART status. Particularly, while being female reduced the odds of having elevated LDL [aOR (95% CI): 0.374 (0.164 - 0.854); P= 0.019], having tertiary education [aOR (95% CI): 3.887 (1.430 - 10.296); P= 0.008], being Ga Adangme [aOR (95% CI): 2.010 (1.011 - 4.363); P= 0.047] , belonging to other ethnic groups [aOR (95% CI): 1.956 (1.083 - 3.532); P= 0.026] and current use of ART [aOR (95% CI): 2.936 (1.661 - 5.189); P= 0.000] increased the odds of having elevated LDL. The likelihood of having low HDL was higher for females [aOR (95% CI): 5.632 (2.724 - 10.874); P= 0.000] and those who were employed [aOR (95% CI): 3.329 (1.065 - 10.404); P= 0.039], compared to their respective counterparts. On the other hand, use and non-use of health insurance ([aOR (95% CI): 0.373 (0.179 - 0.776); P= 0.008] and [aOR (95% CI): 0.582 (0.276 - 0.990); P= 0.047], respectively), being overweight [aOR (95% CI): 0.363 (0.145 - 0.907); P= 0.030] and being on ART [aOR (95% CI): 0.121 (0.061 - 0.238); P= 0.000] reduced the odds of having low HDL. None of the variables were significant predictors of hypertension.

## Discussion

This study was set out to examine the prevalence and potential correlates of cardiovascular risk factors among PLHIV in a tertiary hospital in Ghana. Overall, the study showed that hypertension, diabetes, hypertriglyceridemia, hypercholesterolemia, low high-density lipoprotein, and elevated low-density lipoprotein were predominant among PLHIV. The results further showed that being unemployed, underweight, being on antiretroviral medications, being male, having higher level of education, and not having health insurance subscription significantly increased the odds of these cardiovascular risk factors among the patients.

The prevalence of cardiovascular risk factors among PLHIV is an emerging public health challenge with a higher burden in SSA [20, 21]. Globally, there exists limited studies examining HIV-cardiovascular risk factors comorbidity, and this paucity of knowledge on the topic is more apparent within the context of Ghana where the study was undertaken. The present study revealed a high prevalence of cardiovascular risk factors among PLHIV and this is largely consistent with other studies [5, 6, 22-26]. In Ghana, two studies exist for possible comparison with the hypertension prevalence observed in the present study [17, 18]. While the first study showed comparable hypertension prevalence (9.0%) [17] with our study (9.7%), the second study revealed a higher prevalence of hypertension representing 36.9% of the PLHIV [18].

Similar to hypertension, studies have also reported diabetes to be a key comorbidity among PLHIV [27-29]. Diabetes prevalence among the PLHIV in our study was 15.6%; this is higher than the figure recorded in Ethiopia (12.7%) [23] and Malawi (6.6%) and lower than the rate observed in the Cameroonian context (26%) [30]. The different figures for the prevalence of hypertension and diabetes among PLHIV in these studies may be due to differences in the study designs, sample size, measurements, or contexts. While the prevalence of hypertriglyceridemia and low HDL observed in our study was lower in our study compared to another study in Ghana [17], the proportions of PLHIV with hypercholesterolemia and, elevated LDL were higher in our study.

Generally, our results showed that employment status, current ART status and BMI status were significantly associated with the burden of cardiovascular risk factors among the PLHIV. Our findings showed that cardiovascular risk factors were higher among patients who were unemployed. This is not surprising as research showed that employment loss is frequent during the first years following HIV infection [31]. Our data particularly showed that about 88.0% of those who were unemployed had been living with HIV for 0-5 years. While we cannot determine whether unemployment precedes or occurs after the HIV infection, previous studies have shown a bi-directional association between unemployment and health. That is, individuals with poor health tend to experience unemployment and unemployment has the tendency to lead to deteriorating health [26, 32]. Hence, on the one hand, it is possible that the presence of cardiovascular risk factors may have increased the risk of unemployment among the PLHIV. On the other hand, unemployment may have increased cardiovascular risk factors among our study participants. Even though our data cannot substantiate this time-sequence causation, this may be worth exploring in subsequent studies.

Further, our findings showed that those who were on antiretroviral medications generally had a higher burden of cardiovascular risk factors. This alluded to the fact that even though effective antiretroviral treatment prevents AIDS and non-AIDS related morbidity and mortality, treatment may not fully restore health. Our finding is consistent with several studies which showed higher risk of cardiovascular risk factors among HIV patients on ART [8-12, 14, 18, 33-36]. For instance, research showed that there are complex ways on how ART contributes to cardiovascular risk factors. First, evidence shows that some antiretroviral drugs have direct effects on cardiovascular disease. This is because prolonged exposure to protease inhibitors is associated with hyperlipidaemia, insulin resistance, and a higher rate of cardiovascular disease events [14, 37]. Second, adherence to ART affects virologic response and thus immune activation and is in turn associated with lifestyle and psychosocial NCD risk factors [38]. Further, ART has been causally linked with early onset of cardiovascular diseases even after controlling for the traditional risk factors of NCDs and age [14, 35]. Several cohort studies have shown that even if antiretroviral drugs have no known cardiovascular toxicity, the negative effect of HIV disease on the cardiovascular system may persist during effective treatment [14]. Further, our study showed that those who were overweight or obese had lower odds of having cardiovascular risk factors. This is consistent with what other studies have shown [39].

The findings from this study highlights the growing burden of cardiovascular risk factors among people living with HIV in SSA. The findings reawaken earlier calls by the global health community to repurpose the health systems to respond to the growing burden of NCDs and HIV comorbidity. This is critical in Ghana and other countries in SSA where health systems are constrained and underfunded. Key cardiovascular risk factors such as hypertension, diabetes, hypercholesterolemia, hypertriglyceridemia, elevated LDL, and low HDL were predominant among the population of PLHIV and this mirrors the situation in the global contexts. It is therefore important to prioritise routine screening services, lifestyle modification interventions and emphasis on medication adherence to ameliorate the growing burden of cardiovascular risk factors among PLHIV. In order to improve access to care among PLHIV, integration of healthcare services in health facilities for both HIV and cardiovascular risk factors is critical. Research about the impact of the integrated approach is limited but evidence from Cambodia points to potential improved health outcomes [40]. Further research to explore the feasibility of this strategy and the potential impact in sub-Saharan Africa is recommended. It is also important for longitudinal studies to investigate the potential causative factors for cardiovascular risk factors among PLHIV.

**Limitations:** given that this study was based on an observational cohort study, evidence of associations was only drawn and no evidence about causality was made. As a result, extra caution must be taken in interpreting the study findings. Also, other risk factors such as cell folate deficiency, thyroid dysfunction, hepatitis B and C infection, and paraproteinemia were not considered, and this has the potential to bias the study findings. In addition, since many patients in Ghana usually seek medical attention late, it is likely that the information on the length of HIV infection may be inaccurate. Finally, data for this analysis was based on a single site, and this has implications for representativeness of the evidence, necessitating the need for future studies to involve multiple sites.

## Conclusion

This study highlights the growing burden of cardiovascular risk factors among PLHIV in SSA. The findings indicate that hypertension, diabetes, hypercholesterolemia, hypertriglyceridemia, elevated LDL, and low HDL are predominant among PLHIV at the KBTH, Accra Ghana. Socio-economic factors (such as employment status and level of education), anti-retroviral therapy, and BMI status are key predictors of cardiovascular risk factors in this group. The findings thus emphasise the need to prioritise routine care for PLHIV to minimise cardiovascular risk factors which add extra burden to these individuals and the overall health system. There is the need for a more concerted effort to reform the health systems to respond to the complex health needs of PLHIV. In addition, behavioural interventions are needed to ensure lifestyle modifications in order to prevent cardiovascular risk factors among this population group. The findings further brings into sharp focus the debate about using an integrated approach to ensure routine screening, diagnosis and improved access to care in this population. Given that the findings are from a single site, further multisite studies to advance a broader understanding of the burden of cardiovascular risk factors among PLHIV is warranted.

### What is known about this topic

- Although well-established evidence exists on the burden of NCDs or HIV, evidence on the burden of cardiovascular risk factors among people living with HIV in Ghana is highly under-researched, limited and poorly understood;
- Two studies have been conducted on cardiovascular risk factors-HIV interaction in Ghana; even though they provided important insights on the risks of comorbid conditions among PLHIV, they are limited in scope and geographical coverage.

### What this study adds

- This study shows that there exists a high burden of cardiovascular risk factors among people living with HIV/AIDs (PLHIV);
- Anti-retroviral therapy and BMI increase cardiovascular risk in PLHIV;
- Having higher level of education, being male and being unemployed increase the odds of cardiovascular risk factors among PLHIV.
